# Combined Plasmid Redesign and Transfection Optimization Significantly Increases Upstream AAV Titers While Maintaining Vector Quality and In Vivo Potency

**DOI:** 10.3390/microorganisms14081857

**Published:** 2026-08-20

**Authors:** Shiliang Hu, Yinxing Chen, Carmen Wu, Wilhad Hans Reuter, June Deng, Nannan Jia, Noel Walsh, Amy Bastille, Thomas M. Edwards, Matthias Hebben, Nelson Chau, Jing Liao

**Affiliations:** 1 Genomic Medicine, Alexion, AstraZeneca Rare Disease, 100 Binney Street, Cambridge, MA 02142, USA; 2 LogicBio Therapeutics, 65 Hayden Avenue, Lexington, MA 02421, USA

**Keywords:** AAV production process, two-plasmid system (2P), replication-competent AAV (rcAAV)

## Abstract

A high manufacturing cost of goods (CoG) remains a critical barrier to the broad clinical adoption of gene therapies and is driven in part by limited productivity in adeno-associated virus (AAV) manufacturing. Here, we report an optimized AAV production process developed to markedly increase upstream titers while preserving vector quality and potency. The process combines a redesigned plasmid system, an optimized plasmid ratio, and a novel synthetic transfection reagent and was benchmarked against a conventional triple-plasmid/PEI MAX workflow. Across multiple AAV capsids and independent production runs, the optimized process reproducibly increased crude harvest titers by approximately 10- to 33-fold relative to the standard process, while maintaining key vector quality attributes. Notably, within the detection limits of the assay, rcAAV was undetectable at 1 × 10^10^ vg input with the optimized process, whereas the conventional triple-plasmid (with native Rep-Cap sequence)/PEI MAX workflow remained rcAAV-positive under identical conditions. Importantly, the in vivo potency was comparable to that of vectors produced by the conventional process. These results position our optimized AAV production process as a promising strategy to materially reduce AAV manufacturing CoG per patient.

## 1. Introduction

Adeno-associated virus (AAV) vectors have emerged as one of the most widely adopted delivery platforms for in vivo gene therapy, owing to their favorable safety profile, broad tissue tropism, and sustained transgene expression [1]. However, despite increasing clinical success, the large-scale manufacture of recombinant AAV remains a major bottleneck for the field [2]. Conventional production processes are characterized by low volumetric yields, high cost of goods (CoG), and substantial variability in product quality, particularly when transitioning from laboratory to clinical or commercial scales. These limitations translate directly into restricted patient access, escalating development costs, and challenges in supporting high-dose or systemic indications. As AAV-based therapies expand toward broader patient populations and chronic diseases [3], there is an urgent need for manufacturing innovations that simultaneously increase upstream productivity while maintaining stringent quality and potency requirements [4].

In response to these challenges, extensive efforts across academia and industry have focused on improving AAV production efficiency and robustness. A diverse set of strategies has been explored, including optimization of host cell lines [5,6], refinement of transfection reagents and conditions, enhancement of culture media and feeding strategies [7,8,9], and development of alternative production platforms such as baculovirus–insect cell [10,11] or producer cell systems [12]. In parallel, downstream innovations—ranging from improved affinity ligands to advanced polishing workflows—have contributed to incremental gains in recovery and purity [13]. Although these advances have improved AAV manufacturing, gains in productivity are frequently accompanied by increased process complexity, scalability challenges, or compromises in critical quality attributes [14]. Consequently, integrated upstream solutions that improve productivity without increasing manufacturing complexity remain an important unmet need in AAV process development.

Plasmid and vector design are recognized as key upstream factors affecting AAV manufacturability. Prior studies have demonstrated that the architecture, stoichiometry, and regulatory elements of helper, rep, cap, and gene-of-interest (GOI) expression cassettes can strongly influence viral genome replication, capsid assembly, and packaging efficiency [15,16]. To enhance rAAV yields, various molecular strategies have been reported under specific conditions, including promoter selection and tuning, intron incorporation, codon optimization, and reduction in transcriptional burden [12,17,18,19,20]. Additionally, optimization of plasmid ratios in transient transfection systems has been shown to mitigate imbalances in Rep and Cap expression that can otherwise limit productivity or compromise vector quality [21,22]. Crucially, these early molecular design choices also dictate the generation kinetics of replication-competent AAV (rcAAV), structurally like wild-type AAV, a critical impurity that arises when rep/cap sequences inadvertently undergo homologous or non-homologous recombination with the ITR-flanked transgene cassette [23].

The conventional triple-plasmid (3P) transfection system is widely used but remains constrained by relatively low yields and the need to balance multiple plasmid inputs, factors that can influence intracellular production dynamics and reduce the proportion of full capsids [24]. The presence of multiple independent DNA backbones may also increase opportunities for recombination events associated with rcAAV formation [23]. While some large-scale workflows deconstruct these elements into four-plasmid configurations to independently tune cytotoxic Rep expression relative to structural Cap proteins, the overarching paradigm shift has driven the field toward highly consolidated architectures [25]. This simplification began with early two-plasmid configurations consolidating helper and structural functions [26], followed by helper virus-free, multi-serotype dual-plasmid platforms [27]. More recently, advanced two-plasmid systems have been designed to improve scalability and manufacturing efficiency [28,29], while highly consolidated single-plasmid approaches have further simplified vector production workflows [30].

By physically integrating adenoviral helper, packaging (rep/cap), and transgene-related functions into more-streamlined DNA architectures, these newer platforms reduce the need for manual ratio adjustment, simplify technology transfer, and have been reported to improve volumetric productivity relative to conventional triple-plasmid systems [29,30]. Several studies have also suggested that such configurations may reduce packaging of unwanted plasmid backbone sequences and decrease opportunities for recombination-associated impurities, including rcAAV. Despite these advances, most reported approaches have focused on optimizing plasmid architecture or transfection conditions independently. Whether coordinated optimization of plasmid design and transfection strategy can achieve substantially greater improvements in productivity while preserving vector quality and biological activity remains insufficiently explored.

To address this gap, we investigated an integrated upstream strategy combining rational plasmid redesign with transfection optimization to improve recombinant AAV manufacturing performance. Specifically, we introduce a novel two-plasmid (2P) transfection configuration that consolidates essential adenoviral helper and structural packaging functions while preserving the sequence separation of crucial viral components. When coupled with optimized plasmid ratios and transfection conditions, this approach delivers a 10- to 33-fold increase in upstream AAV titers—a gain demonstrated across multiple serotypes and across escalating production scales while preserving key vector quality attributes and biological activity. In addition, rcAAV was not detected at 1 × 10^10^ vg input in the optimized system, in contrast to the detectable rcAAV observed with the 3P process under the same conditions. By demonstrating that the coordinated optimization of plasmid architecture and transfection parameters can unlock drastic gains in volumetric productivity, this work establishes a robust, highly consistent, and broadly applicable manufacturing framework for gene therapy production platforms.

## 2. Materials and Methods

### 2.1. Plasmid Design and Production Systems

**Figure 1:**
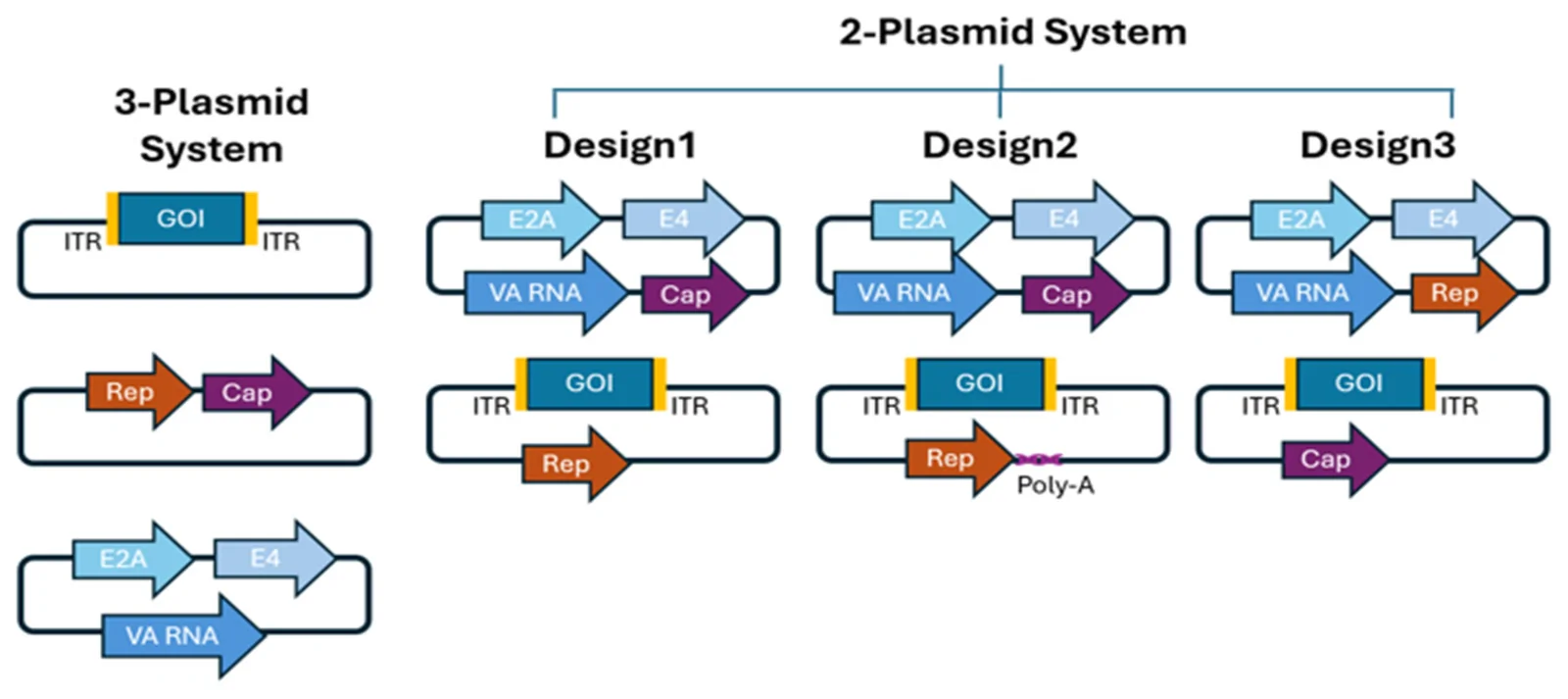
Schematic representation of the conventional 3P and three redesigned 2P system configurations. Two helper plasmids, Helper/Cap and Helper/Rep, and three GOI plasmids, GOI/Rep, GOI/Rep-polyA, and GOI/Cap, were generated. Design 1 is Helper/Cap + GOI/Rep; Design 2 is Helper/Cap + GOI/Rep-polyA; and Design 3 is Helper/Rep + GOI/Cap. LK03 was chosen as Cap in those designs.

To assess vector production efficiency, we compared two adeno-associated virus (AAV) manufacturing platforms: a conventional helper-free 3P system and a consolidated 2P system. Both used the same GOI expression cassette to enable a controlled comparison of packaging efficiency and vector yield. In the 2P system, the ITR-flanked GOI cassette, rep, cap, and adenoviral helper elements were redistributed into two constructs. In Design 1, a Cap/Helper plasmid containing cap and the adenoviral helper unit (E2A, E4, and VA RNA) was paired with a GOI/Rep plasmid containing the ITR-flanked GOI cassette and rep. Design 2 used the same arrangement but added an engineered polyadenylation signal downstream of rep to prevent transcriptional read-through. In Design 3, a Rep/Helper plasmid containing rep and the adenoviral helper unit was paired with a GOI/Cap plasmid containing the ITR-flanked GOI cassette and cap (Figure 1).

Following construction, the three primary 2P configurations were evaluated under identical transfection conditions for AAV production performance. Based on initial screening results, Design 3 was selected for subsequent construct-engineering studies; additional expression characterization studies were subsequently performed using Design 3. To evaluate the impact of intronic elements on rep expression, four Design 3 variants were generated containing either no intron, a 133 bp intron, a 1.4 kb intron, or a 3.3 kb intron immediately upstream of the rep coding sequence. These variants were evaluated under identical conditions to compare AAV production performance and vector quality.

Cloning of all plasmids was performed using standard molecular biology workflows. High-fidelity DNA amplification was performed using Q5 High-Fidelity 2X Master Mix (New England Biolabs, Ipswich, MA, USA, Cat #M0541S) to amplify the rep, cap, and helper elements and the 133 bp, 1.4 kb, and 3.3 kb intronic sequences. Gibson Assembly Master Mix (New England Biolabs, Cat #E2611S) was used for seamless, scarless multi-fragment integration into the backbones. The assembled constructs were transformed into Stbl3 cells (Thermo Fisher Scientific, Waltham, MA, USA, Cat #C737303), followed by plasmid purification via a QIAGEN Plasmid Plus Mega Kit (QIAGEN, Hilden, Germany, Cat #12981) for transfection-grade DNA. Plasmid sequence integrity, assembly accuracy, and structural organization were confirmed by full-length long-read plasmid sequencing using the Plasmidsaurus platform (Plasmidsaurus, Eugene, OR, USA).

### 2.2. Cell Culture Conditions

The Expi293F™ suspension cell line (Thermo Fisher Scientific, Waltham, MA, USA, Cat #A14527) was utilized as the host platform for recombinant AAV production. Cells were maintained in chemically defined, serum-free Expi293 Expression Medium (Gibco/Thermo Fisher Scientific, Waltham, MA, USA, Cat #A1435101). Cultures were maintained at 37 °C and 8% CO_2_ in a humidified shaking incubator using Thomson Optimum Growth Flasks (Thomson Instrument Company, Carlsbad, CA, USA) agitated at 145 rpm. Cells were routinely passaged every 3 to 4 days to maintain continuous logarithmic growth and a baseline viability exceeding 95%.

For vector production experiments, cultures were split 24 h prior to transfection to ensure the population was in the mid-logarithmic growth phase. On the day of transfection, viable cell density and percent viability were assessed using a Beckman Coulter Vi-CELL BLU Cell Viability Analyzer (Beckman Coulter, Brea, CA, USA, Cat #C19201), which automates the trypan blue dye exclusion method to determine cell count, viability, and size. The culture density was adjusted to approximately 2.0 × 10^6^ cells/mL in fresh, pre-warmed Expi293 Expression Medium, ensuring that initial cell viability was maintained at greater than 95%.

### 2.3. Transient Transfection

Transient transfection was performed to directly compare recombinant AAV (rAAV) production efficiencies between the conventional triple-plasmid system and the integrated 2P system. Plasmid DNA was combined using the ratios specified for each experimental condition, while maintaining a total DNA input of 1.0 µg per 1.0 × 10^6^ cells at the time of transfection. To evaluate complexation vehicles, plasmid DNA was complexed using either PEI MAX (linear polyethylenimine; Polysciences, Newtown, PA, USA, Cat #26008-50) or FectoVIR-AAV Transfection Reagent (Sartorius AG, Göttingen, Lower Saxony, Germany, Cat #120100) with a reagent-to-DNA ratio of 1 mL reagent per 1 mg plasmid DNA, both optimized for suspension HEK293-derived cells. For both transfection platforms, the total transfection mixture volume was standardized to 10% (1/10) of the final culture volume, and all polyplex complexation steps were performed at room temperature (20–25 °C) for exactly 30 min prior to delivery.

### 2.4. AAV Production Upstream and Downstream

Seventy-two hours post-transfection, cell cultures were treated with benzonase (100 U/mL) (Sigma-Aldrich, St. Louis, MO, USA, Cat #1016970010) for 15 min at 37 °C to degrade residual plasmid and host cell DNA. Cells were subsequently lysed by addition of lysis buffer containing final concentrations of 10% Tween-20, 500 mM Tris, and 20 mM MgCl_2_ (pH 8.0), followed by incubation for 90 min at 37 °C. Sodium chloride was added to a final concentration of 0.25 M, and lysates were clarified by centrifugation at 3900 rpm for 10 min at room temperature. Clarified supernatants were collected for downstream analyses.

Clarified supernatants were subsequently subjected to downstream purification. Briefly, the AAV-containing harvest was loaded onto an AKTApure system equipped with a CaptureSelect AAV affinity chromatography column (Thermo Fisher Scientific, Waltham, MA, USA, Cat#A36651) to capture viral capsids and remove host cell impurities. The bound AAV particles were eluted according to the manufacturer’s recommended protocol and pooled for further purification.

The affinity-purified material was then processed by cesium chloride (CsCl) density gradient ultracentrifugation to separate full and empty capsids based on their buoyant density. Samples were ultracentrifuged at 50,000× *g* for 18.5 h, and fractions corresponding to genome-containing AAV particles were collected based on density and analytical characterization.

Collected fractions were subsequently buffer-exchanged and desalted using an Amicon centrifugal filter (Sigma-Aldrich, St. Louis, MO, USA, Cat#UFC910008) to remove CsCl and transfer the AAV into the final formulation buffer. The purified AAV preparation was then processed under sterile conditions, aliquoted and stored at −80 °C for long-term preservation until further use.

### 2.5. Product Quality and Analytical Characterization

Recombinant AAV (rAAV) vector titers were quantified using droplet digital PCR (ddPCR) (Bio-Rad Laboratories, Hercules, CA, USA) following nuclease treatment (Thermo Fisher Scientific, Waltham, MA, USA, Cat. # EN0521) and lysis. Vector genomes copy numbers were determined using validated primers and probes targeting the transgene sequence, and titers were reported as viral genomes per milliliter (vg/mL). Vector genome titer served as the primary endpoint for comparing production performance across plasmid configurations, plasmid ratios, and transfection conditions.

In addition to titer determination, vector quality attributes were evaluated using validated analytical characterization methods. Endotoxin levels were measured using a chromogenic LAL assay. Genome integrity and the percentage of full-length transgene were evaluated by alkaline gel electrophoresis of vector genomes. Capsid protein composition and purity were characterized by CE-SDS to assess total capsid purity, VP1:VP2:VP3 ratios, and potential protein aggregation. Residual plasmid DNA was quantified using nucleic acid-based analytical methods.

For process comparisons, vectors generated under different plasmid configurations or transfection conditions were analyzed using an identical analytical workflow to ensure data consistency and comparability across conditions.

### 2.6. Replication-Competent AAV Detection

Replication-competent AAV (rcAAV) testing was performed by Genosafe, Évry-Courcouronnes, France on behalf of LogicBio Therapeutics. HeLa cells were transduced with test vector batches in the presence or absence of wild-type adenovirus and subjected to three successive amplification rounds. Total genomic DNA was extracted after each round and analyzed by real-time qPCR for AAV Rep2 and an endogenous HeLa-cell gene used for cell-number normalization. An increase in Rep2 copies per cell across successive amplification rounds was considered indicative of rcAAV.

### 2.7. In Vivo Studies

To assess functional performance beyond physicochemical comparability, in vivo potency was evaluated in C57BL/6 wild-type mice (*n* = 5 per group; Charles River Laboratories, Wilmington, MA, USA). Animals received a single intravenous tail-vein administration of rAAV vectors produced using either 2-plasmid (2P) or 3-plasmid (3P) systems at an equivalent dose of 1 × 10^13^ vg/kg. Systemic transgene expressions were quantified by measuring circulating human FIX (hFIX) levels at 3, 5, and 7 weeks post-dose using a validated hFIX protein quantification assay. All animal studies were conducted in accordance with protocols approved by the appropriate Institutional Animal Care and Use Committee (IACUC).

## 3. Results

### 3.1. Selected 2P Configurations Increase Productivity and Show Applicability Across Different Serotypes

To reduce the manufacturing CoG, we generated several two-plasmid system configurations in which Helper/Cap or Helper/Rep plasmids were paired with GOI/Rep, GOI/Rep-polyA, or GOI/Cap plasmids (Figure 1). These configurations were tested across multiple ratios, and crude harvest titers (vg/mL) were compared to those collected in the 3P system (Figure S1).

Among the configurations containing Helper/Cap, a 6:1 helper-to-GOI ratio produced the highest titer (Figure S1). At this ratio, Design 1 and Design 2 yielded comparable titers; however, neither configuration resulted in a significant increase relative to the titer obtained with the 3P system. In contrast, Design 3 outperformed both Design 1 and Design 2, with a 1.5:1 helper-to-GOI ratio yielding the highest crude harvest titer of 8.65 ± 0.08 × 10^10^ (Figure S1). Under this condition, viral vector yield increased by approximately 4-fold relative to the 3P control, and the difference was significant. Based on this result, Design 3 was selected as the final configuration of our 2P system. Collectively, these findings indicate that plasmid arrangement substantially influences vector productivity in the 2P system.

**Figure 2:**
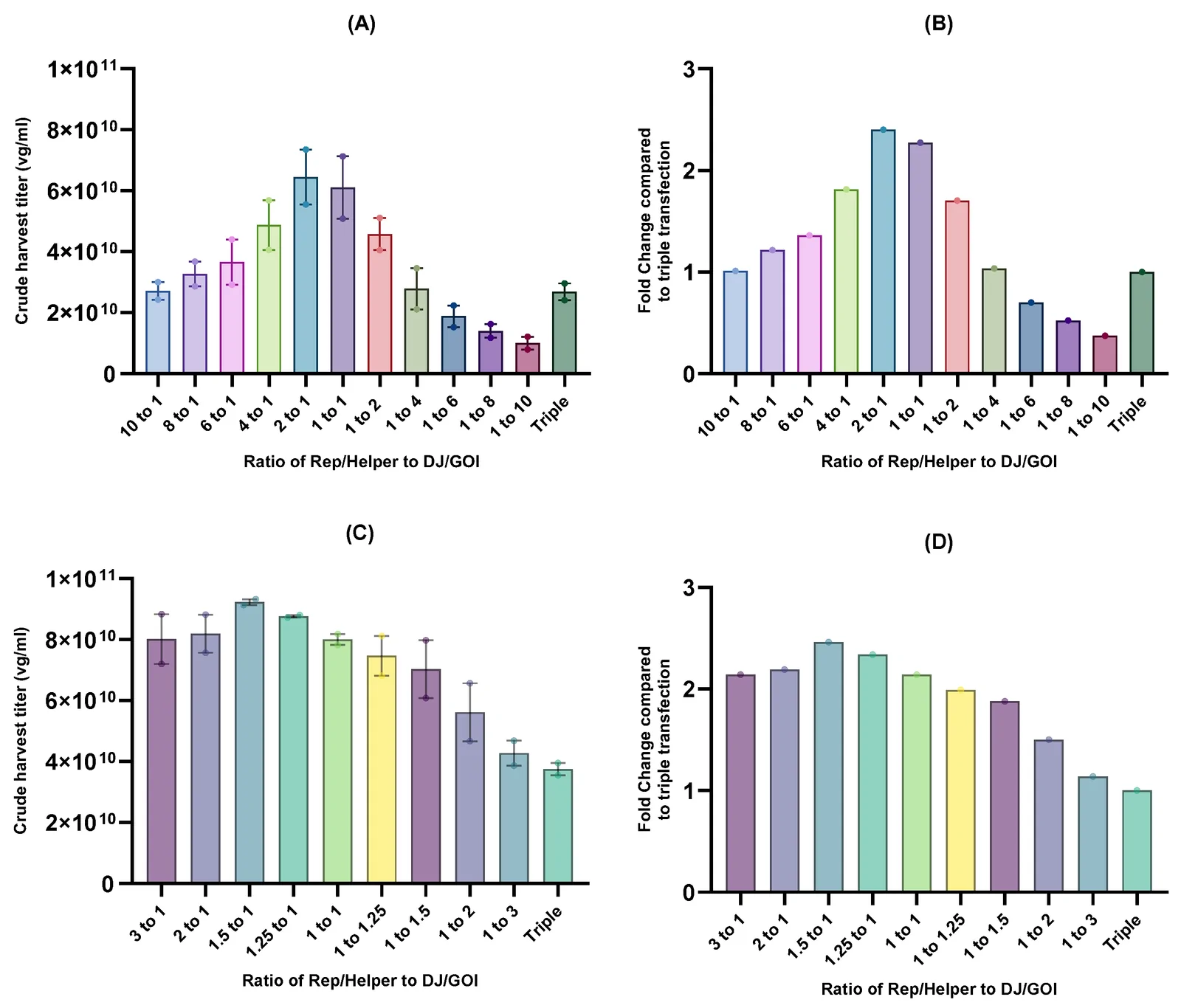
Comparison of two-plasmid and three-plasmid systems for cell transfection. (**A**) Crude harvest titers (vg/mL) generated using different two-plasmid ratios are shown relative to the three-plasmid system. Data are presented as mean ± SD (*n* = 2). (**B**) Relative fold-change in crude harvest titer is shown compared with the three-plasmid system. (**C**) Crude harvest titers (vg/mL) generated using a refined range of two-plasmid ratios are shown relative to the three-plasmid system. Data are presented as mean ± SD (*n* = 2). (**D**) Relative fold-change in crude harvest titer is shown compared with the three-plasmid system. The cap gene encodes AAV-DJ, and the GOI is human Factor IX (hFIX) flanked by murine albumin homology arms (mHAs).

To identify the optimal plasmid ratio for the 2P system, we first performed a broad screen of Rep/Helper to Cap/GOI ratios ranging from 10:1 to 1:10. Crude harvest titers are shown in Figure 2A, and the corresponding fold change relative to the 3P control is shown in Figure 2B. In this initial screen, the 2:1 ratio produced the highest titer, followed by the 1:1 ratio, suggesting that enrichment of Rep/Helper relative to Cap/GOI may improve vector production.

We then refined this screen by testing a narrower range of ratios from 3:1 to 1:3 (Figure 2C). In this follow-up experiment, the 1.5:1 and 1.25:1 ratios yielded higher titers than either the 2:1 or the 1:1 condition, with the 1.5:1 ratio producing the highest overall titer and a 2.46-fold increase relative to the 3P control. These results identified 1.5:1 as the optimal transfection ratio for the 2P system.

**Figure 3:**
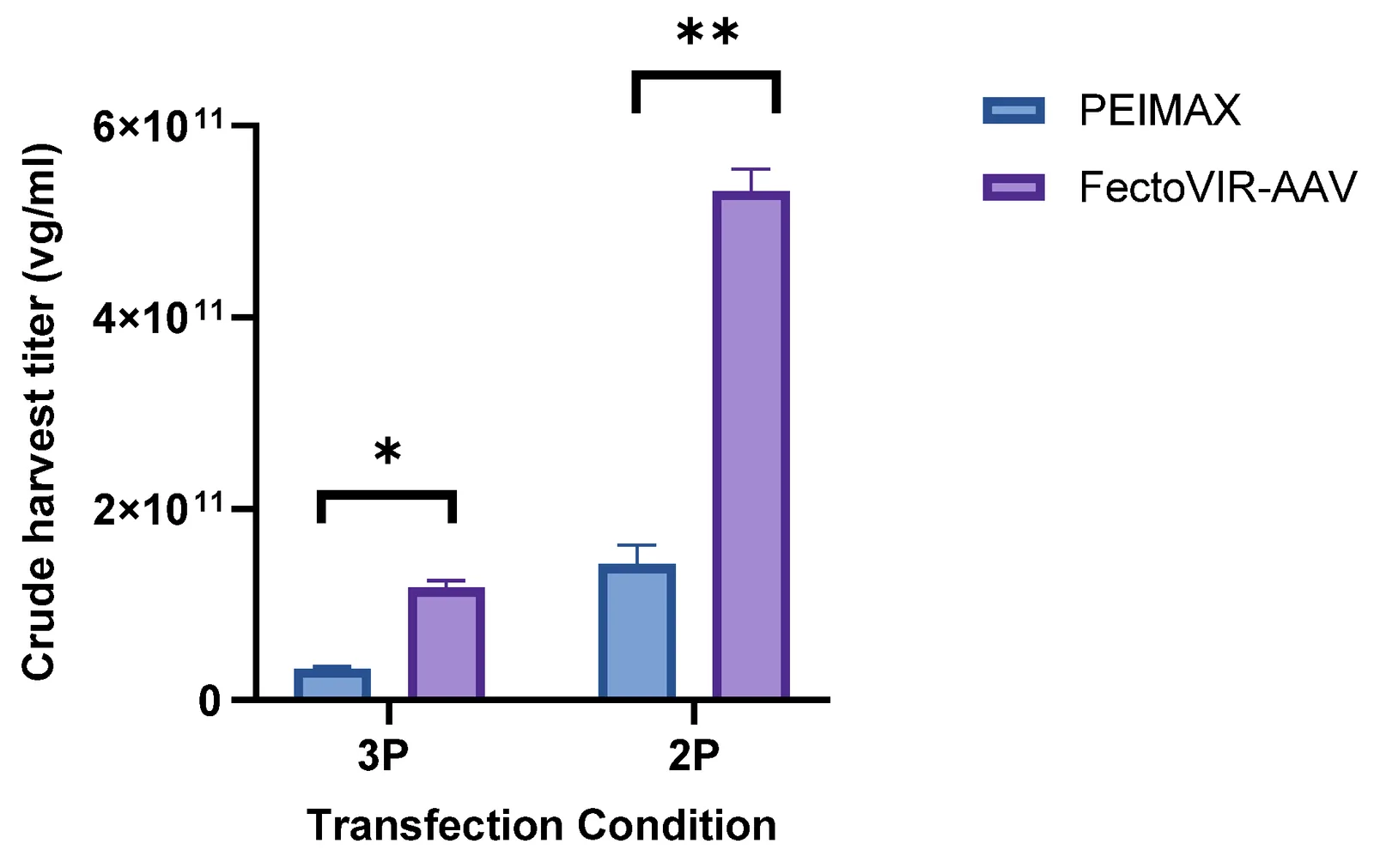
Comparison of 2P and 3P production systems with different transfection reagents. Crude harvest titer (vg/mL) in shake-flask cultures was assessed using an LK03 capsid carrying a human Factor IX (hFIX) GOI, following transfection with PEI MAX or FectoVIR-AAV. Data are presented as mean ± SD (*n* = 2); *: *p* < 0.05, **: *p* < 0.01 (unpaired two-tailed Welch’s *t*-test).

After identifying the optimal 2P configuration and ratio, we evaluated whether the transfection reagent further influenced vector production. PEI MAX and FectoVIR-AAV were compared in both the 3P and 2P systems using LK03 as the capsid and hFIX as the GOI. In both production systems, transfection with FectoVIR-AAV resulted in a marked increase in crude harvest titers relative to PEI MAX. In the 3P system, FectoVIR-AAV increased the titer by approximately 3.6-fold compared with PEI MAX (Figure 3). A similar effect was observed in the 2P system, where FectoVIR-AAV increased the titer by approximately 3.7-fold relative to PEI MAX (Figure 3). In both systems, the increase observed with FectoVIR-AAV was significant. These data indicate that transfection reagent selection has a major impact on vector yield in both 2P and 3P production systems.

Notably, the 2P system transfected with FectoVIR-AAV outperformed the 3P system transfected with FectoVIR-AAV, yielding an approximately 4.5-fold higher crude harvest titer (Figure 3). In addition, the titer increased from 3.29 ± 0.22 × 10^10^ in the 3P + PEI MAX condition to 5.32 ± 0.23 × 10^11^ in the 2P + FectoVIR-AAV condition, representing a 16.2-fold increase (Figure 3). These findings suggest that combining the optimized two-plasmid configuration and plasmid ratio with FectoVIR-AAV can further enhance vector yield.

To assess the broader applicability of the two-plasmid (2P) production system, we evaluated a diverse panel of AAV capsids carrying the same GOI. The capsids tested included AAV2, AAV5, AAV6, AAV8, AAV9, DJ, LK03, and sL65, thereby encompassing both conventional serotypes and engineered capsids. For all comparisons, the 3P system transfected with PEI MAX was used as control. For each capsid, vector production was assessed using both transfection reagents, PEI MAX and FectoVIR-AAV, in combination with both transfection systems, 3P and 2P.

**Figure 4:**
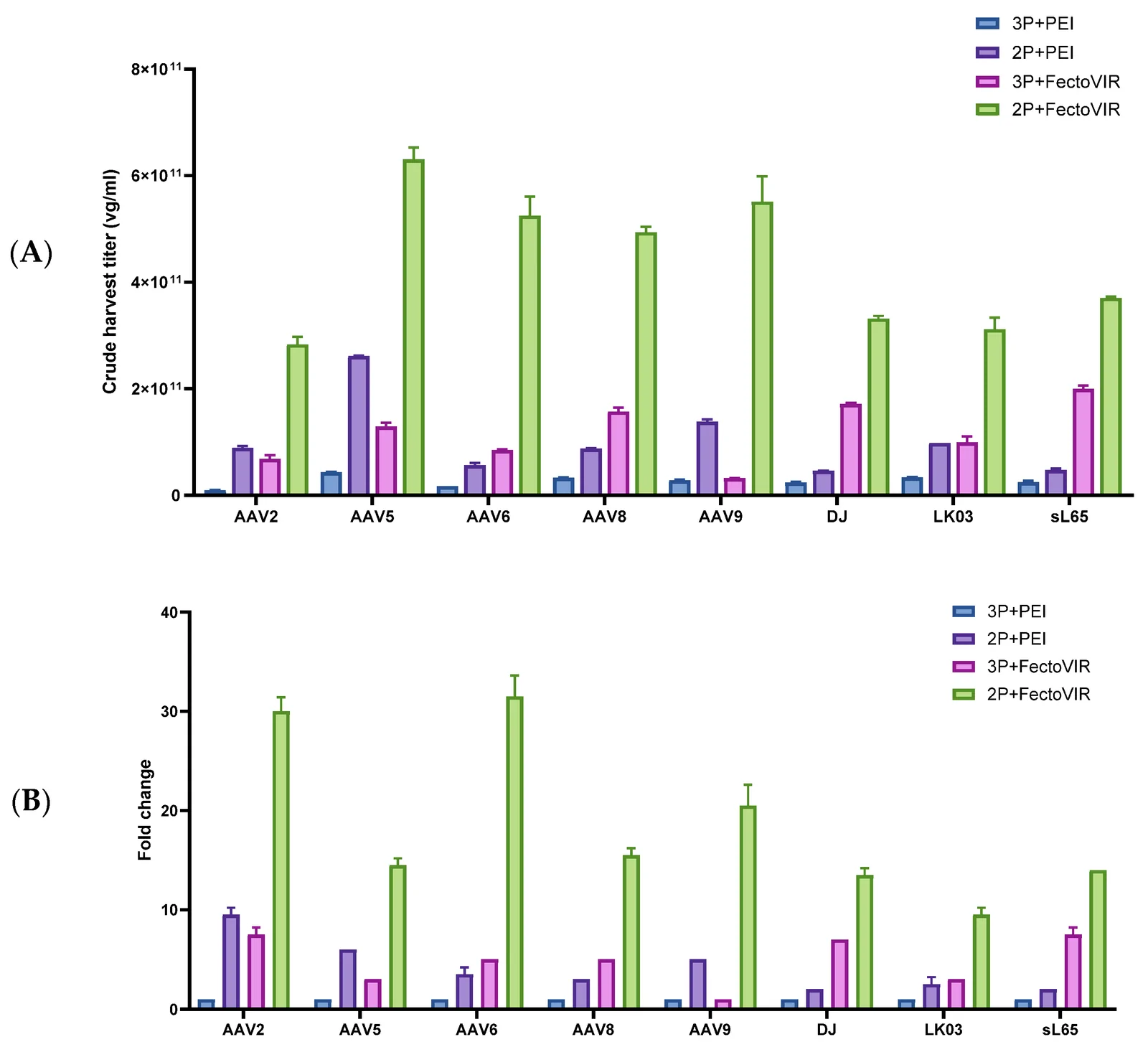
The 2P approach applied to different capsids. (**A**) Crude harvest titers (vg/mL) across several 2P ratios compared with a three-plasmid (3P) system, transfected with PEI MAX or with FectoVIR-AAV. Data are presented as mean ± SD (*n* = 2). (**B**) Fold changes in yield relative to the 3P system. The cap plasmids include AAV2, AAV5, AAV6, AAV8, AAV9, AAV-DJ, AAV-LK03, AAV-sL65 serotypes, and the GOI is human Factor IX flanked by murine albumin homology arms (mHA-hFIX). Data are presented as mean ± SD (*n* = 2).

Across all capsids examined, the 2P system combined with FectoVIR-AAV consistently generated the highest vector titers relative to the 3P PEI MAX control. The next-best performance was observed with either 2P using PEI MAX or 3P using FectoVIR-AAV, depending on the capsid evaluated (Figure 4A). The corresponding fold-change analysis is presented in Figure 4B. Notably, vectors produced using the 2P system transfected with FectoVIR-AAV showed a substantial improvement in productivity, with titers ranging from 10-fold to 33-fold higher than those obtained with the 3P + PEI MAX control. These results demonstrate that the performance advantage of the optimized 2P platform is not restricted to a single capsid type but rather extends across a broad range of natural and engineered AAV capsids.

### 3.2. Assessment of the Quality of the Vectors Produced in 2P System

Although the 2P system improved rAAV production yield relative to the traditional 3P system, vector quality remained a critical consideration. High productivity alone does not ensure an acceptable product profile. Among the potential impurities in rAAV preparations, replication-competent AAV (rcAAV) is a common by-product that occurs during transfection processes.

**Figure 5:**
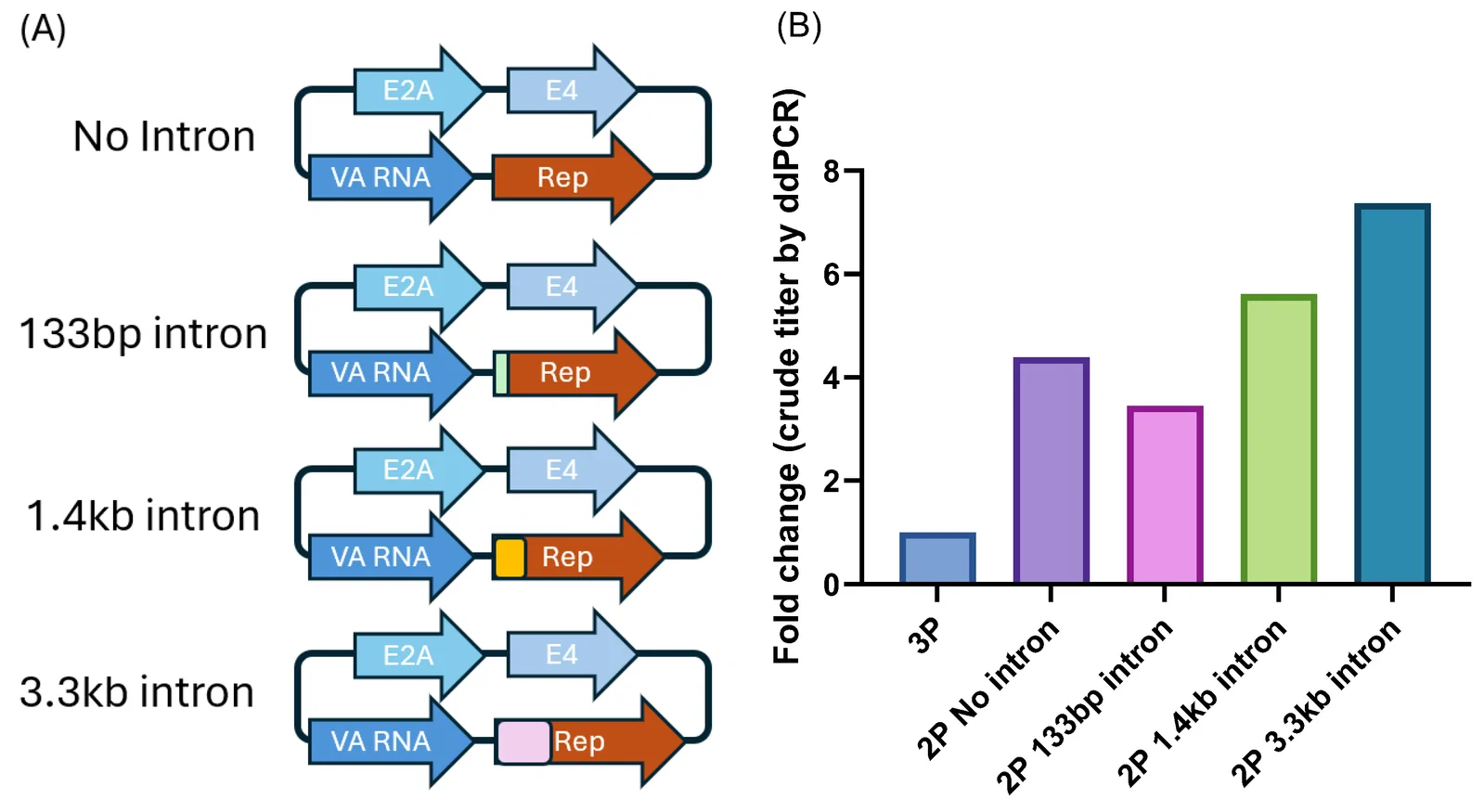
Evaluation of the impact of intron insertion into the helper plasmid on vector yield. (**A**) Schematic of intron insertion into the Rep/Helper plasmid. Introns of three different sizes—133 bp, 1.4 kb, and 3.3 kb—were inserted into the plasmid and are represented by green, orange, and pink, respectively. The construct without an intron served as the control. (**B**) Crude harvest titers (vg/mL) from two-plasmid systems containing an additional intron were compared with those from two-plasmid and three-plasmid systems lacking an intron: 2-plasmid constructs containing a 133 bp intron, a 1.4 kb intron, or a 3.3 kb intron were evaluated. The cap gene encoded LK03.

To reduce the likelihood of rcAAV formation, we generated plasmid designs containing introns of different lengths (133 bp, 1.4 kb, and 3.3 kb) based on the work of Cao et al. (Figure 5A) [31]. We then assessed whether intron insertion affected vector productivity by comparing crude harvest titers of constructs with and without introns (Figure 5B). Intron-containing constructs maintained yields comparable to those of the no-intron construct. Moreover, larger intron inserts were associated with an additional increase in yield (Figure 5B).

We next tested whether intron-containing plasmid designs reduced rcAAV generation using an rcAAV detection assay. Two 2P vectors containing intron insertions in the rep sequence were compared with the conventional 3P control, all carrying the same capsid and GOI. No rcAAV was detected in any group at input levels of 1 × 10^6^ or 1 × 10^8^ vg. At 1 × 10^10^ vg, however, rcAAV was detected in the 3P sample, whereas it remained undetectable in both 2P vectors containing intron insertions within the rep sequence (Table 1). These data suggest that intron incorporation into rep reduced rcAAV formation while preserving vector yield.

**Figure 6:**
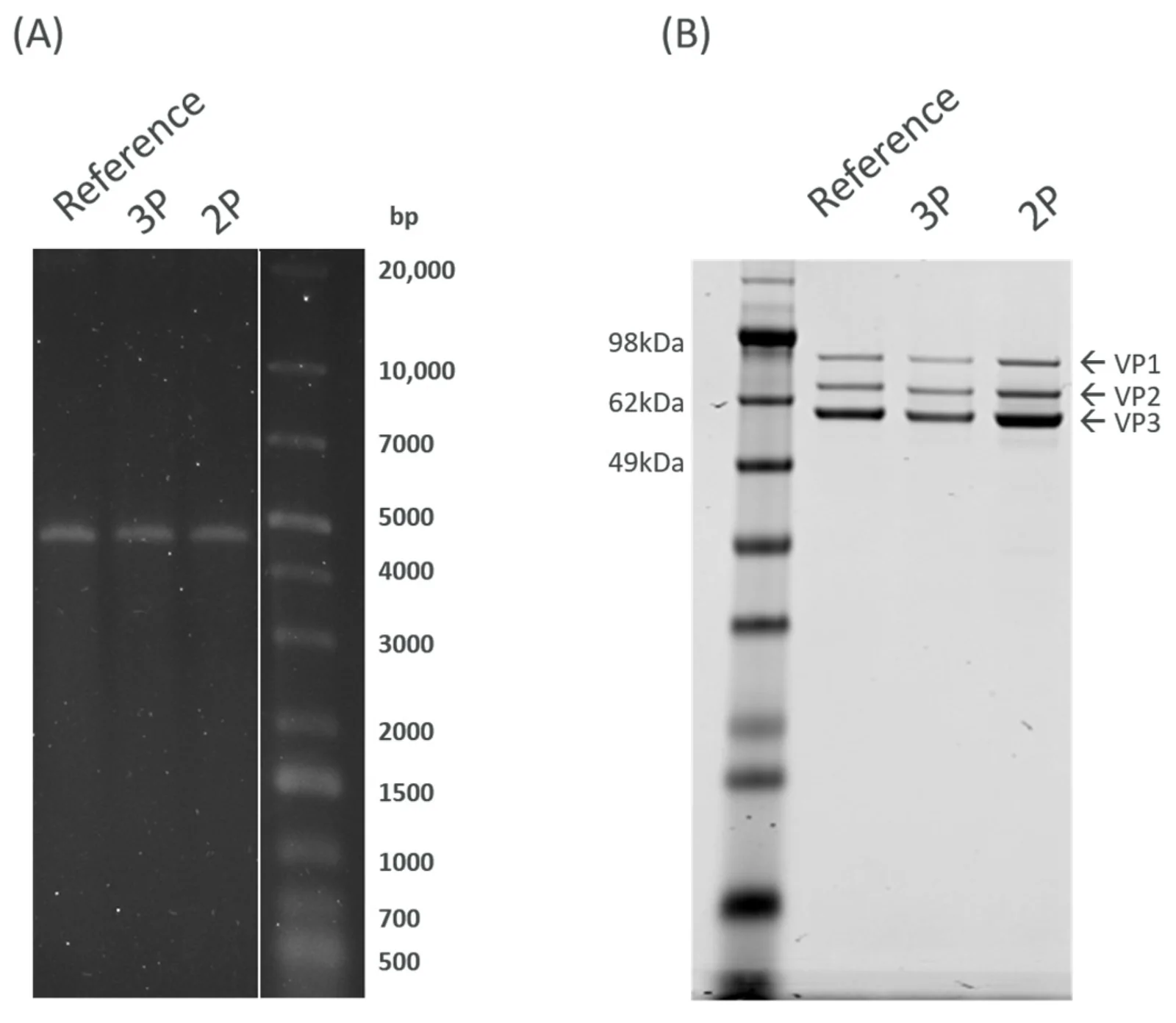
Quality assessment of vectors produced using 3P and 2P systems. (**A**) Alkaline gel electrophoresis analysis of vector genomes. (**B**) SDS-PAGE analysis of vector protein composition.

To further evaluate vector quality, we performed alkaline gel electrophoresis to assess genome integrity in vectors produced by the 2P and 3P systems. In both groups, a single, discrete band was observed that co-migrated with the reference standard and corresponded to the expected transgene size, indicating that the packaged genomes were intact and of the anticipated length (Figure 6A). No obvious evidence of genome degradation, truncation, or aberrant species was detected in vectors generated by either production format. These results demonstrate that the 2P system preserves vector genome integrity at a level comparable to that of the conventional 3P system.

We next examined capsid purity and vector protein (VP) composition by SDS-PAGE. The protein profiles obtained from vectors produced by the 2P and 3P systems showed the expected three major capsid protein bands, corresponding to VP1, VP2, and VP3 (Figure 6B). The overall banding patterns were comparable between the two groups, with no obvious additional bands suggestive of substantial protein impurities, truncated VPs or altered capsid composition. These findings indicate that vectors generated using the 2P system exhibit capsid protein profiles and apparent purity similar to those of vectors produced using the 3P system.

**Figure 7:**
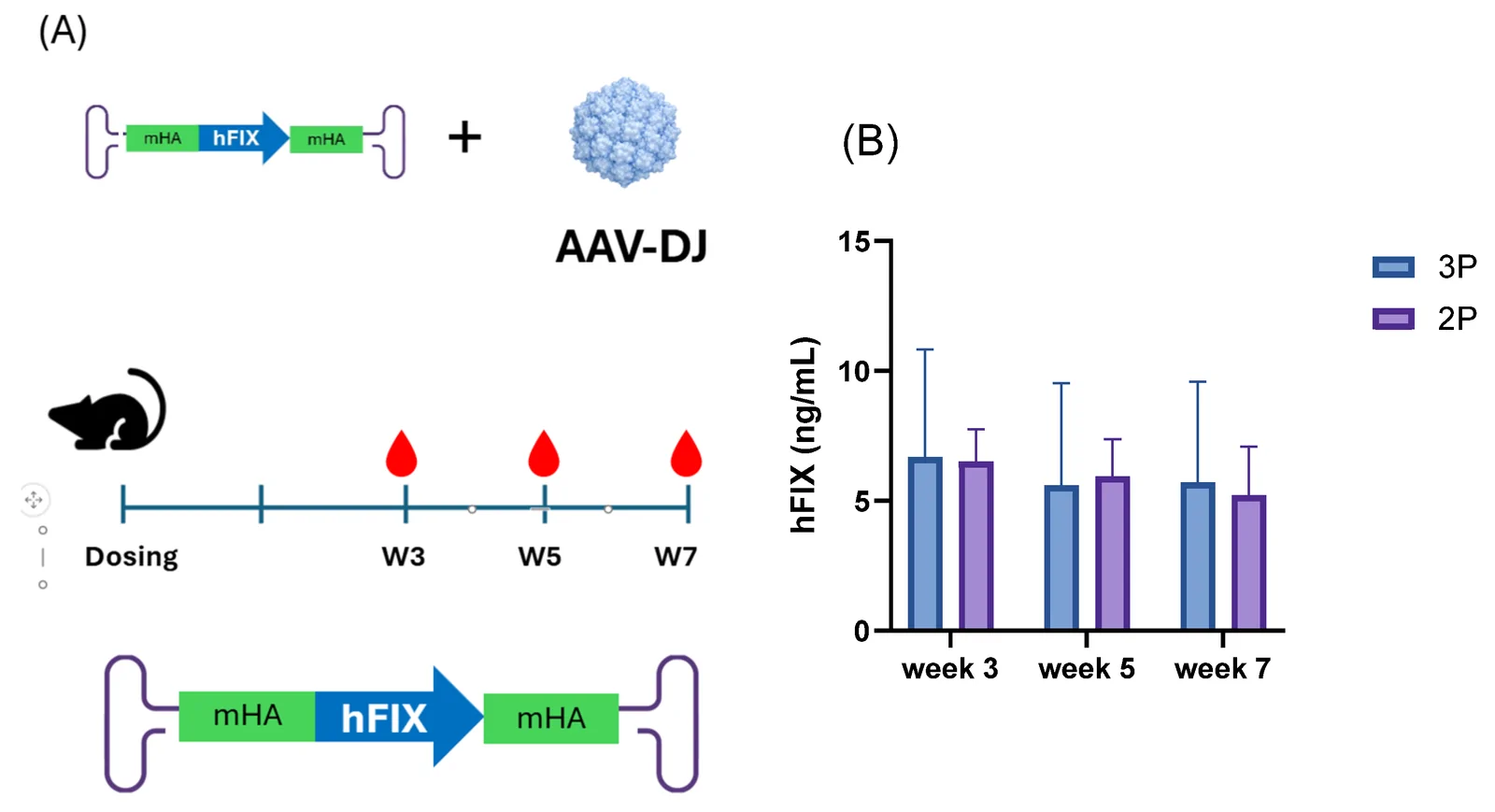
Potency comparison of 3P and 2P. The dosage is 1 × 10^13^ vg/kg. (**A**) Schematic of mouse study. (**B**) The hFIX level is measured 3, 5 and 7 weeks post-dosing. Data are presented as mean ± SD (*n* = 5).

Because physicochemical comparability does not necessarily predict biological performance, we next evaluated the in vivo potency of vectors produced by the 2P and 3P systems. Mice were administered with hFIX vectors at a dose of 1 × 10^13^ vg/kg by intravenous (IV) injection, and hFIX expression was measured at 3, 5 and 7 weeks after dosing (Figure 7A).

Across all three time points, no significant differences in hFIX levels were observed between animals that received vectors produced using the 2P system and those that received vectors produced using the 3P system (Figure 7B). Together, these data indicate that the improved production yield achieved with the 2P system does not compromise vector quality or functional performance and that vectors generated using the 2P format retain in vivo activity comparable to that of vectors produced using the conventional 3P system.

## 4. Discussion

### 4.1. Two-Plasmid System Reduces Manufacturing Cost of Goods

The manufacturing cost of goods (CoG) remains a major barrier to broader patient access in gene therapy, and AAV production continues to be constrained by limitations in scalability, productivity, and process efficiency [1,25,32]. In response to these challenges, extensive efforts across academia and industry have focused on improving AAV production efficiency and robustness. A commonly used strategy is to optimize plasmids, and re-integration of the elements to have fewer plasmids has been attempted [26,27,28,30]. Despite this, determining a reasonable quantity requires weighing various factors. Decreasing the CoG and increasing transfection usually requires a reduction in plasmid numbers. However, integrating one final large plasmid with all elements together ultimately leads to the final construct being more than 20kb; this kind of extra-large plasmid in turn leads to low transfection efficiency [33]. At the same time, this extra-large plasmid can easily cause an instability problem during the scale-up process and also adds some difficulty to molecular cloning [34]. Compared with 4P, conventional 3P and 1P, 2P offers a balanced strategy for simplifying AAV production while maintaining efficiency and stability, reducing process complexity, plasmid DNA demand, and raw material costs, thereby directly lowering the manufacturing CoG.

Although reducing production materials is one way of reducing the CoG, increasing the yield per production unit is a preferred approach. Several 2P designs have been reported. The Grimm laboratory first described the pDG two-vector system, in which the packaging and helper functions are combined on a single plasmid [26,27], and this design was later further refined [28]. However, the original pDG system showed improved yield only relative to the adenovirus-dependent method, whereas the version reported by Tang et al. produced vector yields comparable to those of the conventional three-plasmid (3P) system. Among the three 2P designs evaluated in this study, two did not produce a significant increase in yield. By contrast, Design 3, the Helper/Rep + GOI/Cap configuration, consistently outperformed the 3P system, with the greatest improvement observed at a 1.5:1 plasmid ratio (Figure S1). And this configuration applies to all the serotypes we tested. Especially, it further increases the yield when combined with a novel transfection reagent (FectoVIR-AAV); it achieved a 10- to 33-fold increase in yield compared with the 3P process (Figure 2, Figure 3 and Figure 4). For other serotypes, testing the Rep/Helper-to-Cap/GOI plasmid ratio could help determine the optimal ratio of the two-plasmid system for different serotypes. More importantly, vectors produced using this platform exhibited physicochemical properties and in vivo potency comparable to those of vectors generated using the 3P process (Figure 6 and Figure 7), consistent with previous findings for the 2P design [28]. These results support the robustness and broad applicability of the selected 2P system. Collectively, the findings indicate that how viral functions are distributed across plasmids can substantially affect manufacturing performance, likely by influencing the balance of components delivered during transfection and the efficiency of vector assembly.

From a manufacturing standpoint, the 2P system uses one less plasmid than the 3P system, and its higher productivity may further reduce the cost of goods (CoG) by lowering plasmid demand while increasing process output. In this study, we primarily focused on comparing crude harvest titers under different conditions. The improved upstream yield provides more material for downstream purification, while optimization of the downstream process could help further reduce the CoG. Overall, the combination of reduced plasmid complexity, improved upstream yield, and maintained vector quality and potency highlight the 2P system as a promising platform for more-efficient and economically viable rAAV manufacturing.

### 4.2. Importance of rcAAV Reduction in rAAV Production

Replication-competent AAV (rcAAV) is a recognized critical process-related impurity in rAAV manufacturing [35,36,37]. Although rcAAV has not been shown to cause disease and requires the presence of a helper virus to replicate, its occurrence remains an important product quality concern. It contains rep and cap sequences flanked by inverted terminal repeats (ITRs), enabling replication and packaging when helper functions are available [35,37]. During manufacturing, rcAAV can arise through homologous or non-homologous recombination between the vector genome and packaging components [23,35].

Control of replication-competent AAV (rcAAV) in clinical products is essential not only for regulatory reasons but also to mitigate potential biological and clinical risks. The FDA has noted that expression of Rep and Cap proteins from rcAAV in transduced or infected cells may elicit unintended immune responses, with the potential to compromise therapeutic efficacy [35]. This concern is supported by clinical observations from hemophilia B gene therapy studies, in which AAV capsid-specific CD8+ T-cells responses were associated with loss of transgene expression [38,39] even though the immune response was not directly attributed to rcAAV. Unintended immune responses may be more pronounced in individuals with prior exposure to wild-type AAV. Minimizing rcAAV is therefore an important element of robust AAV manufacturing, and its control will likely become increasingly important as gene therapies are deployed more broadly across larger and more-diverse patient populations.

Multiple strategies have been reported to reduce rcAAV formation, including separating the rep and cap genes into two distinct transcription units, eliminating overlapping sequences among production components, and incorporating heterologous introns into the helper genome together with a mutant AAV terminal repeat that is defective in packaging [23,26,31,36,40]. However, most of these approaches were developed for either 2P with adenovirus helper virus or 3P systems. As noted above, plasmid architecture is a major determinant of both vector productivity and impurity formation, yet strategies to control impurities have often been considered separately from broader upstream process design. In the present study, we implemented an rcAAV mitigation strategy specifically tailored to the 2P platform by redesigning the Rep construct to include intron insertions. This approach differs from that described by Cao et al., which used an intronized helper together with a mutant ITR. In that strategy, because the same type of ITR is present in the vector plasmid, residual homology between the helper constructs and vector plasmids may still permit homologous recombination events that regenerate functional ITRs linked to helper-derived sequences. Although this risk could potentially be mitigated by using heterologous ITRs, our approach relies solely on intron insertion, making it simpler and more readily adoptable. It was designed to reduce unintended packaging of helper-derived rep/cap sequences while preserving vector productivity.

Our data indicates that this redesign improved the impurity profile of the resulting vectors. Vectors produced using the redesigned Rep plasmids showed a marked reduction in rcAAV signal, with rcAAV becoming undetectable at an input level of 1 × 10^10^ vg (Table 1). In contrast, vectors generated using the conventional 3P system remained rcAAV-positive at the same testing level (Table 1). Although published studies have suggested an acceptable level of 1 IU per 1 × 10^8^ vg, rcAAV should ideally be reduced to the lowest level possible [37]. These findings show that the redesigned Rep architecture not only supports efficient vector production but also addresses important safety-related impurity. This point is particularly notable because process intensification strategies are often scrutinized for the possibility that higher productivity may come at the cost of increased impurity burden. In our study, the redesigned 2P system instead coupled higher upstream performance with improved rcAAV control, suggesting that productivity and impurity mitigation need not be opposing objectives. In other words, the optimized 2P system offers a dual advantage: it has the potential to reduce the CoG by improving manufacturing efficiency and to improve product quality by reducing rcAAV. A plausible explanation for the reduction in rcAAV is that intron insertion increases the size of helper-derived rep/cap sequences beyond the AAV packaging limit of approximately 4.7 kb, thereby reducing the likelihood that these sequences are efficiently packaged into capsids [31,41,42].

In the 3P system, rep and cap reside on the same plasmid, whereas in the 2P system used here, they are separated onto different plasmids with no substantial homology. Although this arrangement may already reduce homologous recombination risk, rcAAV can still arise through non-homologous recombination [23]. The redesigned Rep plasmid may therefore provide an additional layer of protection by functionally preventing packaging of rep and cap sequences. More broadly, these data are consistent with the idea that impurity control can be improved through rational sequence and plasmid design rather than relying solely on downstream removal of problematic byproducts after they have already been generated. Under the conditions tested, both the 1.43 kb and 3.3 kb intron-containing designs reduced rcAAV to undetectable levels at 1 × 10^10^ vg, suggesting that either insert length may be sufficient to confer this benefit.

Despite the similar rcAAV outcome observed with both intron-containing constructs, we selected the smaller 1.43 kb intron design for further development. This choice was based on practical manufacturing considerations, as a more compact Rep/Helper plasmid may be advantageous for plasmid stability, production, handling, and routine implementation while still providing the desired improvement in vector purity. It may also reduce unnecessary plasmid size burden during transfection, which could be beneficial for process robustness and scalability, although this point warrants further study. This is consistent with the broader objective of creating a manufacturing platform that is not only safer but also operationally simpler and more cost-efficient.

Together, these findings show that rational plasmid design can improve both manufacturing performance and impurity control within a scalable upstream platform. In this study, the redesigned 2P system, particularly when combined with optimized plasmid ratios, FectoVIR-AAV, and the redesigned Rep plasmid, addressed two major challenges in rAAV manufacturing: high CoG and control of rcAAV as a key safety-related impurity. Across multiple tested capsids/serotypes, the platform increased upstream productivity while reducing plasmid requirements, supporting its broader applicability for improving manufacturing efficiency. Under the serotype and process conditions evaluated for rcAAV, the redesigned system reduced rcAAV to undetectable levels while maintaining vector integrity and in vivo potency. Collectively, these results support the redesigned 2P platform as a promising approach to improve both the economic and quality attributes of rAAV production.

## 5. Patents

The subject matter reported in this manuscript is related to US20230111556A1/US11814642B2, “Manufacturing and use of recombinant AAV vectors,” originally assigned to LogicBio Therapeutics, Inc., and subsequently associated with Alexion Pharmaceuticals following Alexion’s acquisition of LogicBio [43].

## Figures and Tables

**Table 1:** Comparison of rcAAV detection in three-plasmid (3P) and two-plasmid (2P) systems, with or without intron insertion. All vectors contained the same GOI.

Vector	Production System	Intron in Rep Sequence	rcAAV Detection in 1 × 10^6^ vg	rcAAV Detection in 1 × 10^8^ vg	rcAAV Detection in 1 × 10^10^ vg
LK03-MMUT	3 plasmids	None	Not detected	Not detected	Positive
LK03-MMUT	2 plasmids	1.43 kb	Not detected	Not detected	Not detected
LK03-MMUT	2 plasmids	3.3 kb	Not detected	Not detected	Not detected

## Data Availability

The original contributions presented in this study are included in the article/Supplementary Materials. Further inquiries can be directed to the corresponding author.

## Supplementary Materials

The following supporting information can be downloaded at https://www.mdpi.com/article/10.3390/microorganisms14081857/s1: Figure S1: Crude harvest titer (vg/mL) generated using the two-plasmid (2P) configurations were compared with those obtained using the three-plasmid (3P) system.
