# Supplementary material for: Combined Plasmid Redesign and Transfection Optimization Significantly Increases Upstream AAV Titers While Maintaining Vector Quality and In Vivo Potency
Source: Microorganisms. 2026 Aug 20;14(8):1857. doi: 10.3390/microorganisms14081857 (PMC13515234; doi:10.3390/microorganisms14081857)
Supplement: Supplementary file 1 [file microorganisms-14-01857-s001.zip › microorganisms-4497972-supplementary.pdf]

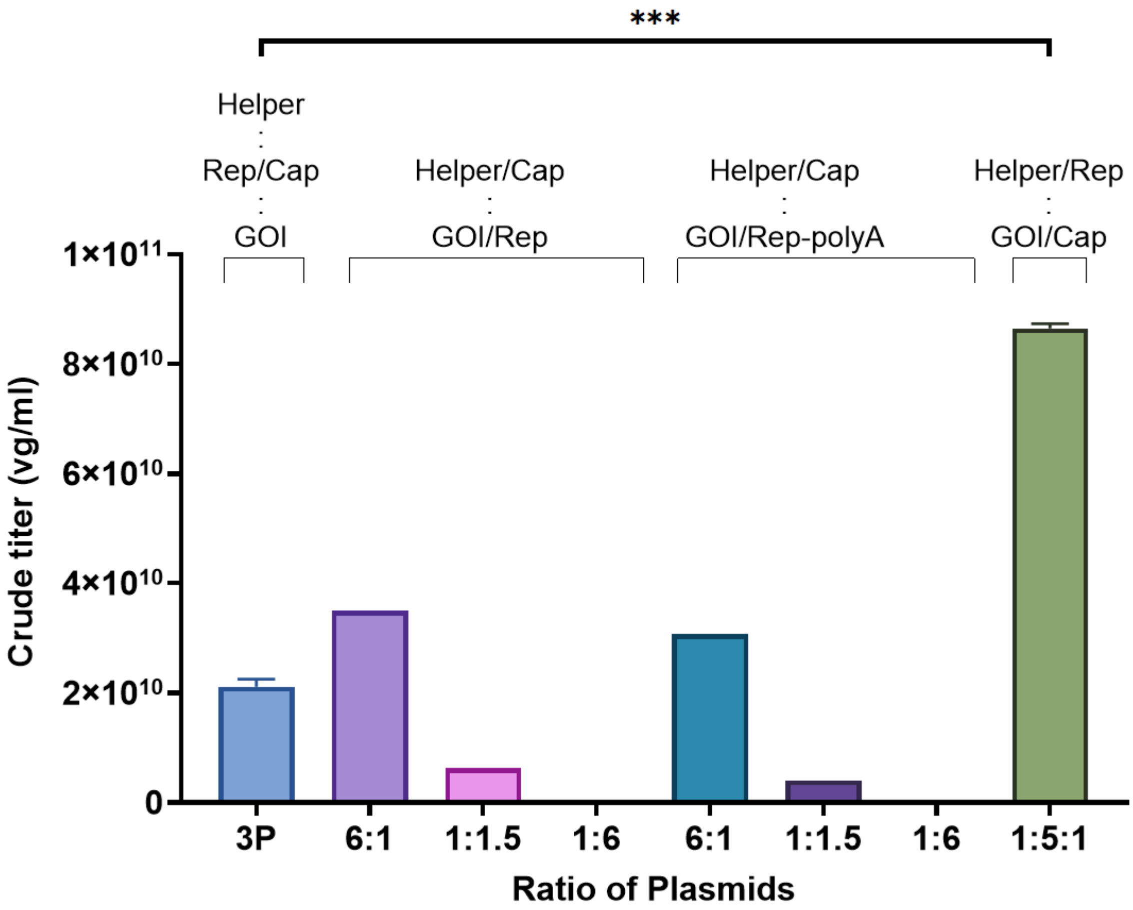

Figure S1. Crude harvest titer (vg/mL) generated using the two-plasmid (2P) configurations were compared with those obtained using the three-plasmid (3P) system. In all conditions, the cap gene encoded the LK03 capsid, and the MMUT GOI was used for transfection. The corresponding plasmid ratios tested are indicated. Data are presented as mean ± SD (n = 2); ***: $p < 0.001$ (unpaired two-tailed Welch's t-test).
